# On-chip photonic decision maker using spontaneous mode switching in a ring laser

**DOI:** 10.1038/s41598-019-45754-3

**Published:** 2019-07-01

**Authors:** Ryutaro Homma, Satoshi Kochi, Tomoaki Niiyama, Takatomo Mihana, Yusuke Mitsui, Kazutaka Kanno, Atsushi Uchida, Makoto Naruse, Satoshi Sunada

**Affiliations:** 1 Graduate School of Natural Science and Technology, Kanazawa University, Kakuma-machi, Kanazawa, Ishikawa 920-1192 Japan; 2 Faculty of Mechanical Engineering, Institute of Science and Engineering, Kanazawa University, Kakuma-machi, Kanazawa, Ishikawa 920-1192 Japan; 3 Department of Information and Computer Sciences, Saitama University, 255 Shimo-Okubo, Sakura-ku, Saitama City, Saitama 338-8570 Japan; 4 Department of Information Physics and Computing, Graduate School of Information Science and Technology, The University of Tokyo, 7-3-1 Hongo, Bunkyo-ku, Tokyo 113-8656 Japan

**Keywords:** Semiconductor lasers, Photonic devices, Nonlinear phenomena

## Abstract

Efficient and accurate decision making is gaining increased importance with the rapid expansion of information communication technologies including artificial intelligence. Here, we propose and experimentally demonstrate an on-chip, integrated photonic decision maker based on a ring laser. The ring laser exhibits spontaneous switching between clockwise and counter-clockwise oscillatory dynamics; we utilize such nature to solve a multi-armed bandit problem. The spontaneous switching dynamics provides efficient exploration to find the accurate decision. On-line decision making is experimentally demonstrated including autonomous adaptation to an uncertain environment. This study paves the way for directly utilizing the fluctuating physics inherent in ring lasers, or integrated photonics technologies in general, for achieving or accelerating intelligent functionality.

## Introduction

In the age of artificial intelligence, the requirements for efficient and intelligent processing of massive amount of data are continuously increasing. Present technologies to accommodate these demands rely on digital electronics; however, hardware scaling in electronics, as foreseen by Moore’s law, is predicted to be unsustainable^1,2^. As a consequence, studies on novel computing principles and architectures beyond the present Turing–von-Neumann computing paradigm^2,3^ are gaining importance. These include neuromorphic computing^4–6^, photonic deep learning^7,8^, reservoir computing^9,10^, molecular computing^11^, and quantum and coherent ising machines^12,13^, where the underlying complexity and fluctuations in natural systems have been used for cognitive processing, prediction, and solving large-scale combinatorial optimization problems.

From the view of information processing, most of the above-mentioned studies have focused on supervised learning or optimization processing. Reinforcement learning is another emergent and important branch of machine learning^14^, where utilization of physical processes can enhance or accelerate their performance^15–18^.

As a foundation of reinforcement learning, decision making plays a key role in engineering applications such as cognitive wireless communication^19,20^, online advertisements^21^, and Monte-Carlo searches^22^. Herein, the decision-making problems under study are called multi-armed bandit (MAB) problems^23^; the goal is to maximize the total reward from multiple slot machines whose reward probabilities are unknown. A key point of the MAB problems is to resolve the *exploration-exploitation dilemma* inherent in decision making under uncertainty: sufficient exploratory actions may inform the best slot machine, but it may be accompanied by a significant amount of losses. In contrast, insufficient exploration may result in missing the best machine.

Recently, we have experimentally revealed that optical fluctuation dynamics can be used for exploring and making an optimal decision in the MAB problems^24–27^. Particularly, it has been found that complex temporal waveforms generated from a chaotic laser are useful for making decisions at a fast rate in the gigahertz regimes^26^. Previous studies suggest the potential of using complex laser dynamics with ultra-wide bandwidth for fast decision making. However, important issues remain open ranging from novel fundamental principles, system architectures, to device implementations for photonic decision making. For instance, the former studies using laser chaos^26,27^ *only* exploit chaotic waveforms as correlated random numbers for a decision making software algorithm; the physical dynamic itself was *not* directly engineered, even though a variety of dynamical features are inherent in laser systems^28^. Furthermore, the previous studies have used a long fiber optic delay line to generate chaotic waveforms^26,27^; such use could lead to impractically large systems, inhibit stable operation, and may prevent practical deployments.

We here propose a compact (<5 mm^2^ area), on-chip photonic decision maker based on a ring laser structure. Unlike previous studies^26,27^, the laser structure can generate fast, complex, but controllable dynamics at a *chip scale*, without a long delay line. The origin of the dynamics is a spontaneous switching phenomenon, i.e., noise-induced mode-hopping^29,30^; the phenomenon is used for exploring an optimal solution under uncertainty. We demonstrate that optimal decision-making is efficiently achieved by opto-electronically controlling the spontaneous-switching dynamics.

## Principle of Ring-Laser-Based Decision-Making

### Ring laser dynamics and device structure

**Figure 1:**
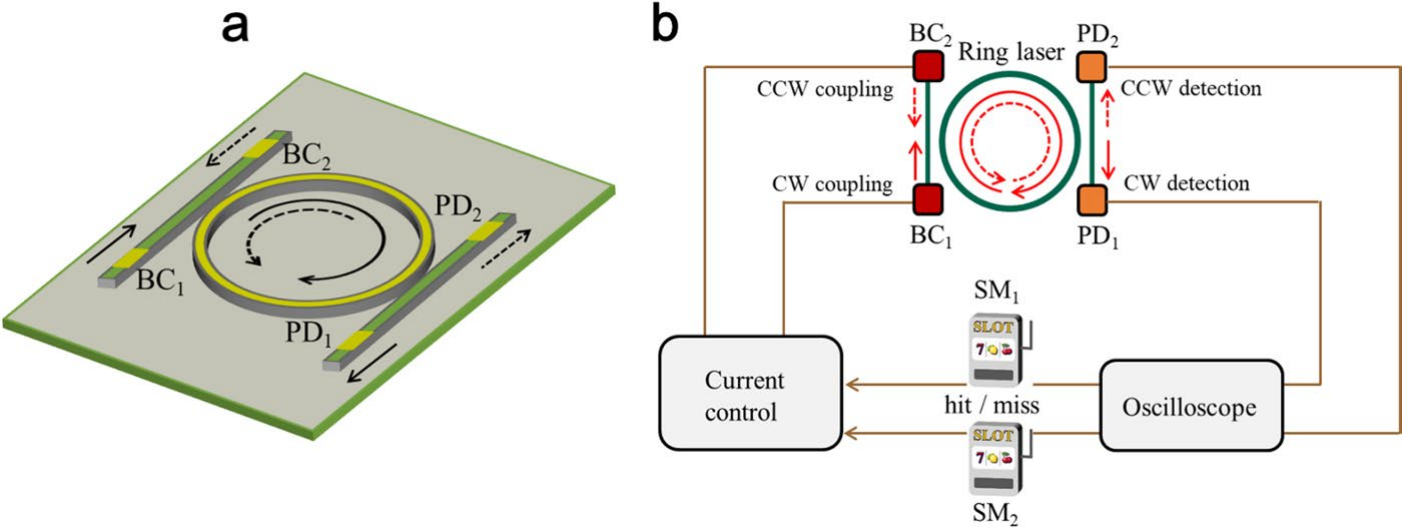
Ring-laser-based decision-making. (**a**) Schematic of the ring laser device coupled to waveguides with contact electrodes BC_*i*_ and PD_*i*_ ($i=1,2$). The ring radius is 1 mm, and the waveguide width is approximately 2 *μ*m. PD_1(2)_ was used as the photodetectors to monitor the CW(CCW) mode intensity in the ring laser, whereas BC_1(2)_ was used for introducing an asymmetry and changing the mode-dynamics. (**b**) Setup for the proof-of-concept experiment on ring-laser-based decision-making. The two PD signals are sent to a digital oscilloscope, and the current is applied to either of BC_1_ or BC_2_ according to the results of the slot machine playing. In the experiment, the slot machines are numerically simulated in the embedded signal processing unit in the oscilloscope.

The device structure used for decision-maker is shown in Fig. 1a. A ring laser is coupled to adjacent waveguides that are integrated on the same chip as a GaAs/AlGaAs single quantum well structure. The resonator of the ring laser supports clockwise (CW) and counter-clockwise (CCW) propagating waves, and can exhibit various operating regimes, such as bidirectional operation and bistability, depending on the pump current^31,32^. Spontaneous switching between the CW and CCW modes is an interesting dynamic that appears in the transition from the stable bidirectional regime to the bistable regime. Spontaneous switching has been regarded as an *obstacle* for deterministic optical switching applications^33–35^. Conversely, in this work, it is preferably utilized for decision making with feedback control of the CW and CCW modes, as discussed later.

The two waveguides with contact electrodes (denoted by PD_*i*_ and BC_*i*_, $i=1,2$, as shown in Fig. 1a, are used for independent input/output control of the two modes in the ring laser: PD_1_ and PD_2_ are used as the photodetectors to monitor the intensities of the CW and CCW modes, whereas BC_1_ and BC_2_ with current injections are used for introducing an asymmetry and changing the dynamics of the CW and CCW modes, respectively^30^. (See *Methods* section for details.) We note that a similar optoelectronic control method has been used for deterministic optical switching^31^ and random number generation^36^. However, unlike the previous studies, we use this method for changing statistical characteristics of spontaneous switching dynamics, as demonstrated later in detail.

### Principle of decision-making

Here, we consider a two-armed bandit (TAB) problem, i.e., the issue is to select the machine with the higher reward probability among two machines, denoted by SM_1_ and SM_2_ (Fig. 1b). We examine a TAB problem, the simplest MAB problem, so that we can validate the principle of the first ring-laser-based decision making. Meanwhile, the scalability of photonic decision making has been studied in the literature^25,27^, which would be applied to ring-laser-based device architectures. Our decision-making method is based on the tug-of-war (TOW) model, exhibiting highly efficient decision making compared to conventional algorithms^15,16^. Based on the model principle, we solve the TAB problem by repeating the following four steps:

(i) **Signal detection**: The intensity level of CW and CCW outputs, denoted respectively by *I*_*CW*_ and *I*_*CCW*_, are detected by photodetector PD_1_ and PD_2_, respectively.
(ii) **Decision of the machine selection**: If *I*_*CW*_ is larger than *I*_*CCW*_, the decision is to select SM_1_. Otherwise, the decision is to choose SM_2_.
(iii) **Playing the selected machine**.
(iv) **Learning and feedback**: If a reward is provided by playing SM_1_ or if a reward is not provided by playing SM_2_, the current (or voltage) applied to BC_1_ is increased to facilitate the lasing in the CW mode. Consequently, the probability of selecting SM_1_ slightly increases in the next decision making. On the other hand, if a reward is provided by playing SM_2_ or if a reward is not provided by playing SM_1_, the current (or voltage) applied to BC_2_ is increased so that the CCW lasing is facilitated, leading to a slight increase of the probability of choosing SM_2_ in the next step.

Repeating steps (i)–(iv), we can finally choose the best slot machine.

As described in step (iv) above, an important point for the decision making is how to change currents *J*_1_ and *J*_2_ to activate controllers BC_1_ and BC_2_, respectively. In this study, we control *J*_1_ and *J*_2_ by the following rules in which a dimensionless, time-dependent control parameter *C*(*t*) is introduced:

1 $$\begin{array}{lll}{J}_{1}=KC(t), & {J}_{2}=0, & {\rm{if}}\,C(t)\ge 0,\\ {J}_{1}=0, & {J}_{2}=-\,KC(t), & {\rm{if}}\,C(t)\le 0,\end{array}$$

where *K* is a gain parameter. If $C\ge 0$ at the *t*-th play, the current ${J}_{1}=KC$ (mA) is injected to controller BC_1_ whereas ${J}_{2}=-\,KC$ is injected to BC_2_ if $C < 0$. The amount of *C*(*t*) is updated in accordance with the results of slot machine playing as follows:

2 $$C(t+1)=\alpha C(t)+{\rm{\Delta }}C,$$

and

3 $${\rm{\Delta }}C=\{\begin{array}{cc}+{\rm{\Delta }} & {\rm{i}}{\rm{f}}\,{{\rm{S}}{\rm{M}}}_{1}\,{\rm{w}}{\rm{i}}{\rm{n}}{\rm{s}}\\ -{\rm{\Delta }} & {\rm{i}}{\rm{f}}\,{{\rm{S}}{\rm{M}}}_{2}\,{\rm{w}}{\rm{i}}{\rm{n}}{\rm{s}}\\ -{\rm{\Omega }}{\rm{\Delta }} & {\rm{i}}{\rm{f}}\,{{\rm{S}}{\rm{M}}}_{1}\,{\rm{f}}{\rm{a}}{\rm{i}}{\rm{l}}{\rm{s}}\\ +{\rm{\Omega }}{\rm{\Delta }} & {\rm{i}}{\rm{f}}\,{{\rm{S}}{\rm{M}}}_{2}\,{\rm{f}}{\rm{a}}{\rm{i}}{\rm{l}}{\rm{s}},\end{array}$$

where $\alpha \in [0,1]$ is the memory parameter (typically, ≈0.99–0.999)^37^, and Δ is an incremental parameter (${\rm{\Delta }}=1$ in this study). ${\rm{\Omega }}$ in Eq. (3) is determined based on the estimated reward probability ${\hat{P}}_{i}$ for SM_*i*_ ($i=1,2$) from the history of the betting results. ${\hat{P}}_{i}$ is given by *L*_*i*_/*S*_*i*_, where *S*_*i*_ is the total number of times of playing SM_*i*_ and *L*_*i*_ is the number of wins in selecting SM_*i*_. ${\rm{\Omega }}$ is then given as,

4 $${\rm{\Omega }}=\frac{{\hat{P}}_{1}+{\hat{P}}_{2}}{2-({\hat{P}}_{1}+{\hat{P}}_{2})}.$$

The details of the derivation of Eq. (4) are shown in^15^.

## Results

### Optoelectronic control of spontaneous switching dynamics

**Figure 2:**
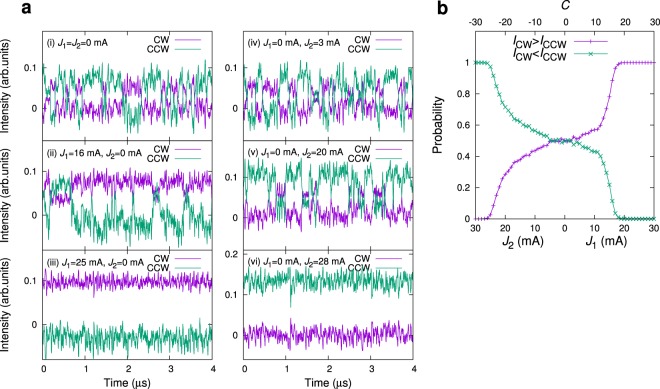
Spontaneous switching dynamics. (**a**) Temporal waveforms of the CW intensity *I*_*CW*_ and CCW intensity *I*_*CCW*_ for various values of currents *J*_1_ and *J*_2_. (**b**) Probability *P*_*CW*_ of ${I}_{CW} > {I}_{CCW}$ (purple color) as a function of currents *J*_1_ and *J*_2_ and the corresponding control parameter *C* with *K* = 1. For reference, the probability of ${I}_{CCW} > {I}_{CW}$, $1-{P}_{CW}$, is indicated by the green color.

In our ring laser, a spontaneous switching phenomenon used for the above decision-making method appears when the pump current *J*_*p*_ exceeded ~1.3 times of the laser threshold current *J*_*th*_. Figure 2a shows the examples of the switching dynamics, where the CW and CCW intensities stochastically change due to internal laser noise. For convenience, we hereafter refer to the state of ${I}_{CW(CCW)} > {I}_{CCW(CW)}$ as the CW (CCW) mode. A statistical analysis reveals that the mode switching is characterized by a characteristic time ${\tau }_{c}$ ≈ 43 ns; in a timescale longer than ${\tau }_{c}$, the switching process is treated as a Poisson (random) process, and the duration time in the CW (CCW) mode obeys an exponential distribution (see Supplementary Fig. A1 for details). We refer to ${\tau }_{c}$ as the correlation time of the switching process. When current *J*_1_ to BC_1_ increases with *J*_2_ = 0, the duration time in the CW mode increases [Fig. 2a(i,ii)]. In particular, we found that for ${J}_{1} > 20\,{\rm{mA}}$, the duration time diverges, and a stable CW mode operation is achieved [Fig. 2a(iii)]. Otherwise, increasing *J*_2_ can lead to an increase of the duration time in the CCW mode [Fig. 2a(iv,v)], and a stable CCW mode operation is achieved for *J*_2_ > 25 mA [Fig. 2a(vi)].

### On-chip decision making: proof-of-concept demonstration

We conducted decision-making experiments based on the controllable dynamics in the ring laser by repeating the processes (i-iv) described in the previous section. In the experimental setup shown in Fig. 1b, the two machines SM_1_ and SM_2_ were emulated in a computer with the reward probabilities of (*P*_1_, *P*_2_) = (0.7, 0.3). The gain *K*, step Δ, and memory parameter *α* were set to be 1, 1, and 0.99, respectively. A machine is selected and played once, and the reward dispensed from SM_1_ and SM_2_ is assumed to be both 1. The goal of the experiment is to confirm whether the ring-laser-based decision maker selects SM_1_ (rather than SM_2_) since SM_1_ has a higher reward probability (*P*_1_ > *P*_2_). We assume the situation of zero prior knowledge, where the sum of the two hit probabilities is unknown, unlike ref.^26^.

**Figure 3:**
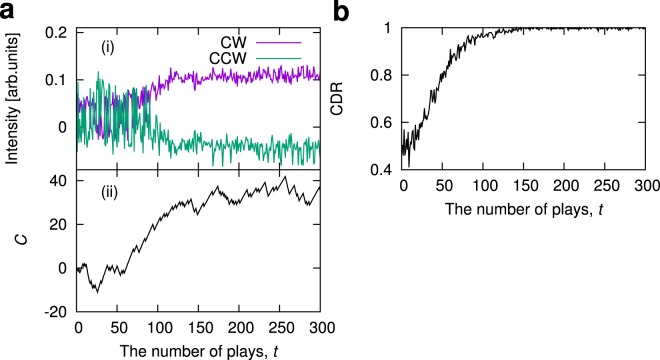
Experimental demonstration of ring-laser-based decision-making. (**a**) Evolution of (i) *I*_*CW*(*CCW*)_ and (ii) control parameter *C* for a single trial. In this experiment, the reward probabilities of SM_1_ and SM_2_ are set as (*P*_1_, *P*_2_) = (0.7, 0.3). *K* = 1, *α* = 0.99, and Δ = 1. (**b**) Evolution of CDR. The CDR was evaluated with *n*_*T*_ = 200 trials.

The experimental results on the decision-making process are displayed in Fig. 3. At first, *I*_*CW*_ and *I*_*CCW*_ are randomly switched when the number of plays *t* < 100 [Fig. 3a(i)], suggesting the exploration to choose the best machine. The accumulated knowledge is used for estimating the reward probabilities and setting the ${\rm{\Omega }}$-value, and then the *C*-value is appropriately updated [Fig. 3a(ii)]. The updated *C*-value affects the dynamics, and the dynamical state change from the switching mode to the CW mode. Consequently, the best machine (SM_1_ in this case) is selected. We repeated the decision-making experiment ${n}_{T}=200$ times and evaluated the correct decision rate (CDR), which is defined as the ratio of the number of selecting the slot machine with higher reward probability at the *t*-th play in *n*_*T*_ trials^24^. As shown in Fig. 3b, the CDR monotonically increases and approaches 1, suggesting the achievement of correct decision making.

**Figure 4:**
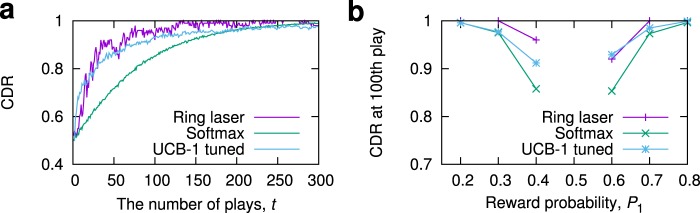
Performance comparison. (**a**) Comparison of CDRs with the modified Softmax and UCB1-tuned. The reward probabilities are set as (*P*_1_, *P*_2_) = (0.6, 0.4). The CDR of the UCB1-tuned is better than the ring laser-based method for the first few ten plays, but the CDR of the ring laser-based method more quickly approaches closely to 1 even before the 100th play. In this experiment, *K* = 4, *α* = 0.99, and Δ = 1 were used. The parameters used in the modified softmax are similar to those in ref.^16^. The UCB1-tuned is a non-parameter algorithm^38,39^. (**b**) The CDRs at the 100th play. Here, *P*_2_ was set to be 1 − *P*_1_.

We also conducted similar decision-making experiments with respects to different reward probabilities and parameters; we found that with appropriately tuned parameters (*K* and Δ), the decision-making performances could be comparable to existing decision-making algorithms such as a modified softmax^16^ and upper confidence bound 1-tuned (UCB1-tuned)^38,39^. As shown in Fig. 4, the CDR of the ring laser-based method can exceed those of the other methods in some cases.

## Discussion

### Decision-making strategy and its control

In our decision-making method, the strategy for making good decisions is characterized by the probability function of inducing CW mode configured by the control parameter *C*(*t*), denoted by *P*_*CW*_(*C*). As observed in Fig. 2b, *P*_*CW*_(*C*) of the ring laser has a plateau region in the range of around −21 ≤ *C* ≤ 12, where *P*_*CW*_(*C*) moderately changes when *C*-value is changed. The plateau region plays a role in explorations to estimate the reward probability (and hence an appropriate ${\rm{\Omega }}$-value), and can lead to a correct decision after many slot plays, as demonstrated in Fig. 4; however, it may also lead to a slow convergence of CDR. A better alternative strategy (i.e., the design of *P*_*CW*_(*C*)) satisfying both fast adaptation speed and decision accuracy can theoretically be estimated in the case when we can obtain prior knowledge on the sum of the reward probabilities, *P*_1_ + *P*_2_, such as when either of two events inevitably occurs with the probabilities *P*_1_ and ${P}_{2}=1-{P}_{1}$.

Let us here assume that the value of *P*_1_ + *P*_2_ is *a priori* known and ${\rm{\Omega }}$ in Eq. (4) is a constant value. For simplicity, we consider *α* = 1 and assume that the mode switching is random. Under these assumptions, we can treat the time evolution of *C* as a random walk. The random walk model gives an analytical expression of CDR and suggests that fast and correct decision is made when the probability distribution *P*_*CW*_(*C*) is close to 1 for *C* > 0 and 0 for *C* < 0, and steeply vary from 0 to 1 near *C* = 0. (See Sec. 2 of Supplementary Information).

**Figure 5:**
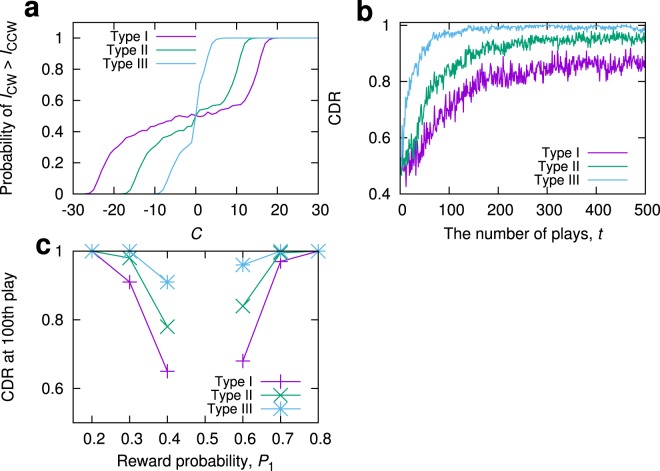
Decision-making strategy. (**a**) C-dependence of the occurrence probability of the state ${I}_{CW} > {I}_{CCW}$. Types I, II, and III represent *P*_*CW*_(*C*) obtained for (*K*_1_, *K*_2_) = (0, 0), (5, 9), and (13, 17), respectively. (**b**) Time evolution of CDR for each type. In this experiment, the reward probabilities were set as (*P*_1_, *P*_2_) = (0.6, 0.4), and prior knowledge of ${P}_{1}+{P}_{2}=1$ was assumed. As predicted by the random walk model, the CDR for Type III is superior than the other types. *K* = 1, *α* = 0.99, and Δ = 1. (**c**) The CDRs at the 100th play were compared as a function of the given reward probability, where Type III outperforms other cases.

In an actual experiment, such a *P*_*CW*_(*C*) is effectively realized by modifying the relationship between the control parameter *C* and *J*_1(2)_ (Eq. 1) as follows:

5 $$\begin{array}{lll}{J}_{1}=0, & {J}_{2}=0, & {\rm{if}}\,C(t)=0,\\ {J}_{1}=KC(t)+{K}_{1}, & {J}_{2}=0, & {\rm{if}}\,C(t) > 0,\\ {J}_{1}=0, & {J}_{2}=-\,KC(t)+{K}_{2}, & {\rm{if}}\,C(t) < 0,\end{array}$$

where *K*_1_ and *K*_2_ are chosen such that the plateau region of *P*_*CW*_(*C*) shown in Fig. 2b is reduced and the desirable *P*_*CW*_(*C*) results. Figure 5a shows *P*_*CW*_(*C*) with (*K*_1_, *K*_2_) = (0, 0), (5, 9) and (13, 17), depicted by Types I, II, and III, respectively. As predicted by the random walk model, CDR in Type III most quickly increases and the convergence value is higher than the other types, regardless of the reward probabilities *P*_1_ and *P*_2_ (Fig. 5b,c). Thus, we conclude that the decision-making performance can be enhanced by changing the intrinsic characteristics (*P*_*CW*_(*C*)) of the physical devices with an appropriate mode-control.

### Decision-making rate

The rate of decision-making, i.e., the number of decision-making per unit time, in principle, depends on the sampling rate of the CW- and CCW-signal detections. Thus, fast decision making is possible by increasing the sampling rate; however, sampling too rapidly may degrade the accuracy of the decision making because nearly identical signal levels will be observed due to the limitation of the ring laser dynamics. It is important to know how rapidly decision making can be made without degrading the performance. In order to address this question and obtain an insight into the effect of the switching dynamics on the decision-making performance, we numerically examine decision-making processes by standard ring laser model equations^32^. See *Methods* section for details of the modeling.

**Figure 6:**
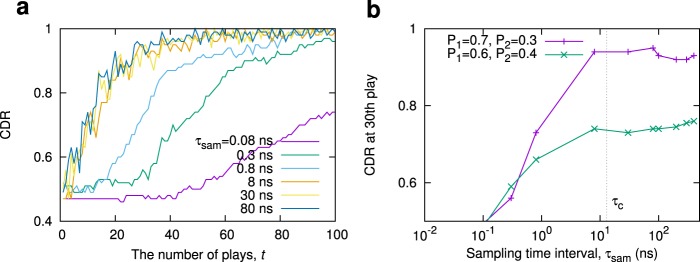
Ultimate operation rate analysis of ring-laser-based decision making. (**a**) Time evolution of CDRs with respects to different sampling time interval ${\tau }_{sam}$. Too small ${\tau }_{sam}$ or too fast operation degrades CDRs. The reward probabilities were set as $({P}_{1},{P}_{2})=(0.7,0.3)$. The CDRs were evaluated from the results of *n*_*T*_ = 100 trials. (**b**) Comparison of CDR at the 30th play as a function of ${\tau }_{sam}$. The reward probabilities were set as $({P}_{1},{P}_{2})=(0.7,0.3)$ and (0.6, 0.4). In this simulation, the correlation time ${\tau }_{c}$ was ≈13 ns, which is indicated by dotted line. We can clearly observe that the CDRs are degraded in the regime where ${\tau }_{sam}$ is smaller than ${\tau }_{c}$.

Figure 6a shows the evolution of the CDR for various values of the sampling rate 1/${\tau }_{sam}$ when (*P*_1_, *P*_2_) = (0.7, 0.3), where is the sampling time interval of the signal detections. The CDRs at the 30th-play are shown as a function of ${\tau }_{sam}$ in Fig. 6b. These numerical results clearly show that the decision-making performance (accuracy and adaptation) degrades when ${\tau }_{sam}$ is much shorter than the correlation time ${\tau }_{c}$ of the ring laser. Actually, the autocorrelation of the switching signals sampled at ${\tau }_{sam}\ll {\tau }_{c}$ exhibits a positive value [See Supplementary Fig. A1(d)]. In the decision-making, the positive correlation may result in repetition of the same choice even when the choice is wrong. In contrast, when ${\tau }_{sam}\gg {\tau }_{c}$, the correlation becomes close to zero, which enables an exploration without repeating wrong choices. Accordingly, the sampling time interval (i.e., inverse of the decision-making rate) can be shorter up to the correlation time without degrading the performance. The correlation time can be shorter in principle, allowing faster decision making by increasing the noise strength and activating mode-hopping phenomenon. In an actual experiment, this can be achieved by coupling the laser to an external amplified spontaneous emission noise source; the experimental verification will be an interesting future study.

## Summary

In this study, we proposed and experimentally demonstrated on-chip photonic decision making by an integrated ring laser. Ring lasers generate statistical characteristics regarding the CW and CCW lasing, which are optoelectronically controllable; we directly utilize such inherent spontaneous dynamics of ring lasers for decision-making functionalities. Correct decision making was successfully demonstrated with appropriate optoelectronic control of the dynamics, and it is found that the performance can be enhanced by changing the decision-making strategy with the statistical characteristics (*P*_*CW*_(*C*)). These results would open novel research perspectives of controlling complex dynamics based on environmental changes.

One interesting and important future study is to increase the decision-making rate by using faster and more complex switching dynamics. In addition to the above-mentioned method on increasing the noise strength, the use of the delayed feedback structure will be useful. Interestingly, semiconductor ring lasers can exhibit chaotic switching in the GHz regimes by delayed feedback even with *a short time delay*^40,41^. Combination of noise-induced switching with delayed feedback instability indicates a promising research direction.

As for the ring laser structure, we emphasize that in addition to miniaturization, it would be beneficial for all optical realization of decision-making devices because all photonic components required for decision making can be monolithically integrated on a chip. Instead of the optoelectronic control methods employed in the present study, it would be interesting to use an optical injection method because ring lasers subjected to optical injection enable low power and ultrafast switching at picosecond time scales^33–35^.

Another interesting and important future study is to tackle larger-scale MAB problems. MAB problems can be solved based on a hierarchical TOW principle^25,27^. The decision-making based on the hierarchical principle can be achieved by using a number of independent two-choice decision-makers (for two-armed bandit problems) or using a time-division multiplexing scheme^27^. Compact ring lasers could offer a good experimental platform for implementing the hierarchical principle and addressing the MAB problems.

We believe that the combination of photonic integration technologies and competitive fluctuating dynamics, as demonstrated by the proposed ring laser, will shed light on a way toward novel photonic intelligent computing paradigms.

## Methods

### Device structure and operating regime

The ring laser device used in this study was fabricated in a graded-index separate-confinement-heterostructure (GRIN-SCH) single-quantum well GaAs/Al_*x*_Ga_1−*x*_As structure, the emission wavelength of which is designed to be 850 nm. The fabricated laser device was thermally controlled by a heat-sink with an accuracy of 0.01 °C. The ring radius is 1 mm, and the waveguide width is 2 *μ*m. In an actual device, multiple waveguides with independent electrical contacts are coupled to the ring with an angle to the cleaved facet. We used the waveguides with contacts, PD_*i*_ and BC_*i*_ ($i=1,2$), as shown in Fig. 1a. The CW and CCW intensity signals are detected with PD_1_ and PD_2_ in the waveguide, respectively, and sent to a digital oscilloscope (Tektronix TDS7154B, bandwidth 1.5 GHz, 20 GSample/s) via the bonding wires attached to PD_1_ and PD_2_. Bias contacts BC_1_ and BC_2_ were used for the mode-control inside the ring laser. Sending current to BC_1_ and BC_2_ reduces the absorption loss of the waveguide. Thus, the light coupled from the CCW(CW) mode in the ring to the waveguide is back-reflected at the BC_1(2)_-side end of the waveguide and re-coupled to the ring in the CW(CCW) direction. In addition, BC_1(2)_ can enhance the spontaneous emission noise coupled to the CW(CCW) mode, and consequently, facilitates the laser operation in the CW(CCW) mode^31,36^.

When ${J}_{1}={J}_{2}=0\,{\rm{mA}}$, the threshold current *J*_*th*_ of the ring laser used in the experiment was approximately estimated to be 210 mA at 25 °C. The large threshold may partly be attributed to non-optimal etching depth of the ring waveguide^32^. For *J*/${J}_{th} < 1.3$, the ring laser operated on a bidirectional state of the CW and CCW modes. For larger *J*-value, a transition to spontaneous switching regime occurred.

### Intensity adjustment

In the experiment, the PD couplings to the CW and CCW modes are not essentially equal to each other due to an imperfect device fabrication. In order to reduce the effect of the asymmetry of the PD-couplings and appropriately evaluate the decision-making performance, the CW and CCW intensities, *I*_*CW*_ and *I*_*CCW*_, were adjusted by adding constant biases so that the occurrence probability is calibrated being around 0.5 when ${J}_{1}={J}_{2}=0\,{\rm{mA}}$. This way would realize easy tuning of both intensities, while we should also note that there is another simpler way, which is to measure either of *I*_*CW*_ or *I*_*CCW*_ only and adjust the switching probability to 0.5 by bias currents *J*_1_ and *J*_2_ without the intensity biases.

### Decision-making experiment

First, the BC_1_ and BC_2_ were connected to a standard current source. Discrete-valued electrical currents were applied to BC_1_ or BC_2_. Then, the CW and CCW optical intensity signals for different values of *J*_1_ and *J*_2_ were recorded by a digital oscilloscope. In the decision-making experiment, the slot machines were numerically simulated in the embedded signal processing unit in the oscilloscope using pseudorandom numbers. The decision is immediately made based on the sampling. The controllers BC_1_ and BC_2_ were also connected to a two-channel function generator (Tektronix AFG3152C), which reconfigures the oscillation dynamics of the ring laser in an on-line or real-time manner.

### Rate-equation model for semiconductor ring laser

The numerical simulation was conducted by using a set of dimensionless semiclassical equations for the two slowly varying complex amplitudes of CW and CCW waves, *E*_1_ and *E*_2_^32^.

6 $$\frac{d{E}_{1,2}}{dt}=(1+i\tilde{\alpha })\,[{\xi }_{1,2}N-1]\,{E}_{1,2}-{k}_{1,2}{E}_{2,1}+{\eta }_{1,2}(t),$$

7 $${\xi }_{1,2}=1-s|{E}_{1}{|}^{2}-c|{E}_{2}{|}^{2},$$

where $\tilde{\alpha }$ accounts for phase-amplitude coupling, *s* and *c* are the self- and cross-saturation coefficients, and *k*_1,2_ represents the complex backscattering coefficients. We model internal optical noises as complex Gaussian noise satisfying $\langle {\eta }_{i}\rangle =0$ and $\langle {\eta }_{i}(t){\eta }_{j}^{\ast }(t^{\prime} )\rangle =2D{\delta }_{ij}\delta (t-t^{\prime} )$ ($i=1,2$). $\langle \cdot \rangle$ represents the ensemble average, and *D* represents the noise strength. Carrier density *N* obeys the following equation:

8 $$\frac{dN}{dt}=2\gamma \,[\mu -N\,(1-{\xi }_{1}|{E}_{1}{|}^{2}-{\xi }_{2}|{E}_{2}{|}^{2})],$$

where *μ* is the dimensionless pumping power ($\mu =1$ at the laser threshold). In the above equations, *t* is dimensionless time rescaled by photon lifetime ${\tau }_{p}$. *γ* is the ration of ${\tau }_{p}$ to carrier lifetime ${\tau }_{s}$.

In Eq. (6), the asymmetric coupling caused by activating BC_1_ and BC_2_ is simply modeled as an asymmetric backreflection effect such that ${k}_{1}={\beta }_{1}{k}_{b}$ and ${k}_{2}={\beta }_{2}{k}_{b}$, where *k*_*b*_ denotes the backreflection coefficient when $C=0$, and *β*_1,2_ denotes a dimensionless asymmetry parameter, depending on *C* as follows:

9 $$\begin{array}{lll}{\beta }_{1}=1+kC(t), & {\beta }_{2}=1, & {\rm{if}}\,C(t)\ge 0,\\ {\beta }_{1}=1, & {\beta }_{2}=1-kC(t), & {\rm{if}}\,C(t)\le 0,\end{array}$$

where *C*(*t*) is updated by Eq. (2). This is the simplest model of the asymmetric backscattering, although a real asymmetry may be introduced in a more complex way in the actual experiment. We confirmed that regardless of the details of the asymmetry model, the control of spontaneous switching can be achieved. The detailed investigation using more realistic model will be a future work.

In this study, we set some of the parameters as follows: $\tilde{\alpha }=3.5$, $2s=c=0.006$, ${k}_{b}=0.004+i0.001$, $k=0.025$, $D=5\times {10}^{-5}$, $\mu =2.0$, ${\tau }_{p}=10\,{\rm{ps}}$, $\gamma =0.01$. With these parameter values, we obtained stochastic switching dynamics with the correlation time ${\tau }_{c}\approx 13\,{\rm{ns}}$ when $C=0$. In the decision-making simulation, we assume that the slot machines provide a reward without any time delay and use Eqs (2–4) and (6–9).

## Supplementary information

Supplementary information
