# Supplementary material for: On-chip photonic decision maker using spontaneous mode switching in a ring laser
Source: Sci Rep. 2019 Jul 1;9:9429. doi: 10.1038/s41598-019-45754-3 (PMC6603012; doi:10.1038/s41598-019-45754-3)
Supplement: Supplementary file 1 — Supplementary information [file 41598_2019_45754_MOESM1_ESM.pdf]

Supplementary information: On-chip photonic decision maker using spontaneous mode switching in a ring laser

Ryutaro Homma[1], Satoshi Kochi[1], Tomoaki Niiyama[1,2], Takatomo Mihana[3], Yusuke Mitsui[3], Kazutaka Kanno[3], Atsushi Uchida[3], Makoto Naruse[4], and Satoshi Sunada[1,2]

[1] *Graduate School of Natural Science and Technology, Kanazawa University, Kakuma-machi, Kanazawa, Ishikawa, 920-1192, Japan*

[2] *Faculty of Mechanical Engineering, Institute of Science and Engineering, Kanazawa University, Kakuma-machi Kanazawa, Ishikawa 920-1192, Japan*

[3] *Department of Information and Computer Sciences, Saitama University, 255 Shimo-Okubo, Sakura-ku, Saitama City, Saitama, 338-8570, Japan*

[4] *Department of Information Physics and Computing, Graduate School of Information Science and Technology, The University of Tokyo, 7-3-1 Hongo, Bunkyo-ku, Tokyo 113-8656, Japan*

1 Duration time statistics

In this section, we analyze the mode switching process shown in Fig. 2a in the main text and show the statistical characteristics for the duration time in each mode.

Figure A,1(a-c) show the probability distributions of the duration time in the CW and CCW modes, which clearly obey exponential distributions for a time scale longer than a characteristics time τ_c, regardless of the J_1- and J_2-values. The exponential distribution of the duration time suggests that the switching is a Poisson process with a long timescale. However, for a short timescale of $\tau < \tau_c$, the distributions deviate from the exponential distributions, suggesting a dynamical feature of the switching process in the short timescale. Actually, as shown in Fig. A,1d, the autocorrelation of the switching signal given by $s = I_{CW} - I_{CCW}$ is positive for $\tau < \tau_c$ and exponentially decays as $\exp(-\tau/\tau_c)$ with the time τ_c. We estimated τ_c as 43 ns from the fitting with the exponential function. In the main text, τ_c has been referred to as the *correlation time.*

We measured the mean duration time in each mode for different values of J_1- and J_2-values. The results are plotted in Fig. A,1(e). One can see that when $J_{1(2)}$ increases, the mean duration time in the CW(CCW) mode increases while the mean duration time of the CCW(CW) mode decreases.

2 Random walk model of decision-making

In this section, we consider a random walk model of decision-making and discuss the design of $P_{CW}(x)$ for better decision-making. According to Eqs. (2)–(3) in the main text, control parameter C takes a step of amount $\pm\Delta$ or $\pm\Omega\Delta$ depending on four events: either of SM_1 or SM_2 is selected and the selected machine wins or misses. Here let Δt be a time interval for the update of C, and $\alpha = 1$ is assumed. Under the assumptions, Eq. (2) in the main text is similar to that of a random walker who takes a step of amount $\pm\Delta x$ or $\pm\Omega\Delta x$ in a time interval of Δt. Hereafter, let x be a position of the random walker. Then, let $\pi_1^{+(-)}(x)$ be the transition probability when the walker at position x selects $SM_{1(2)}$ and wins the reward. Likewise, let $\pi_2^{+(-)}(x)$ be the transition probability when the walker at x selects $SM_{2(1)}$ and

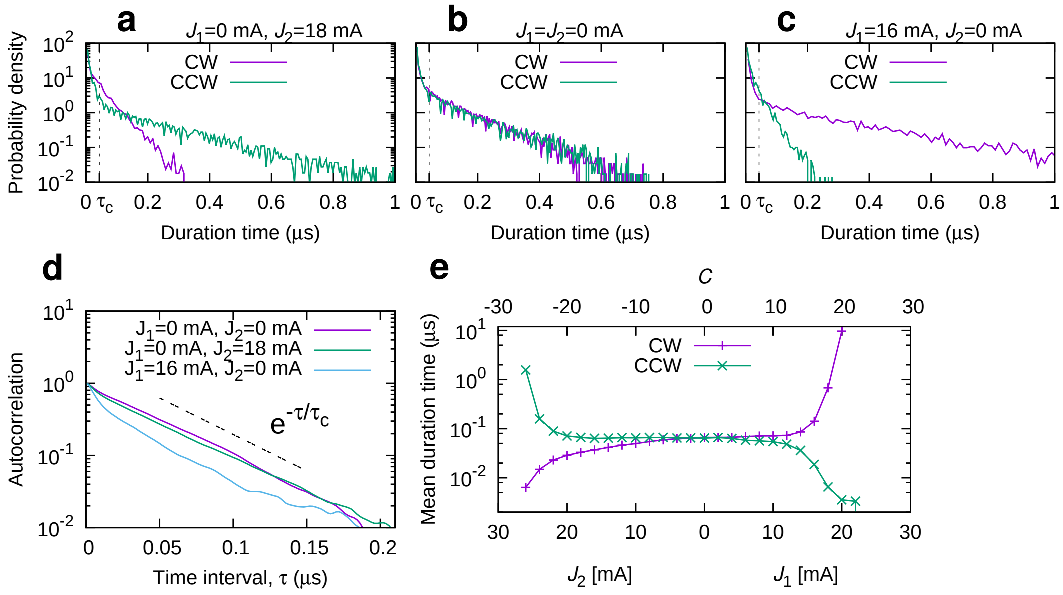

Figure A,1: **Duration time statistics. a, b,** and **c**, Probability densities of the duration time in the CW and CCW modes. **d,** The autocorrelation of the switching signal $I_{CW} - I_{CCW}$. In **a-d**, τ_c represents the correlation time. In the ring laser used in this study, τ_c was estimated to be 43 ns by fitting the autocorrelation functions with the exponential function $\exp[-\tau/\tau_c]$. **e,** The mean duration times as a function of currents J_1, J_2 and corresponding C-value with $K = 1$.

misses the reward. These transition probabilities are given as follows:

$$\pi_1^+(x) = P_1 P_{CW}(x), \quad \text{(B,1)}$$

$$\pi_1^-(x) = P_2\left[1 - P_{CW}(x)\right], \quad \text{(B,2)}$$

$$\pi_2^+(x) = (1 - P_2)\left[1 - P_{CW}(x)\right], \quad \text{(B,3)}$$

$$\pi_2^-(x) = (1 - P_1)\, P_{CW}(x). \quad \text{(B,4)}$$

We are interested in the probability $f(x,t)\Delta x$ that a walker arrives at x in time t. Denoting by $x_k = k\Delta x$ ($k \in \mathcal{Z}$) the discrete position of the walker and approximating Ω as floor(Ω), the following equation holds for the probabilities,

$$\begin{aligned} f(x_k, t + \Delta t) &= f(x_k, t) + \sum_{j=1}^{2}\left[\pi_j^+(x_k - \Delta_j) f(x_k - \Delta_j, t) + \pi_j^-(x_k + \Delta_j) f(x_k + \Delta_j, t)\right] \\ &- \sum_{j=1}^{2}\left[\pi_j^+(x_k) + \pi_j^-(x_k)\right] f(x_k, t) \\ &= \sum_{j=1}^{2}\left[\pi_j^+(x_k - \Delta_j) f(x_k - \Delta_j, t) + \pi_j^-(x_k + \Delta_j) f(x_k + \Delta_j, t)\right], \end{aligned} \quad \text{(B,5)}$$

where $\Delta_1 = \Delta x$, $\Delta_2 = \Omega\Delta x$. In the derivation, $\sum_j(\pi_j^+ + \pi_j^-)$ =1 was used.

The correct decision rate (CDR) can be expressed with the probability $f(x,t)\Delta x$. When SM_1 is the best machine with the highest reward probability, i.e., $P_1 > P_2$, the correct decision rate is written as:

$$\mathrm{CDR}_1(t) = \sum_k P_{CW}(x_k) f(x_k, t)\Delta x. \tag{B,6}$$

Likewise, the correct decision rate for $P_1 < P_2$ is given as

$$\mathrm{CDR}_2(t) = \sum_k \left[1 - P_{CW}(x_k)\right] f(x_k, t)\Delta x. \tag{B,7}$$

Fokker-Planck approximation

In the above model, we can choose arbitrary small incremental quantities, Δx and Δt. Here we assume that Δx and Δt are so small that the Taylor expansion for the Fokker-Planck type equation can be applied to the discrete random walk model. This allows us to gain a physical insight into the decision-making and analyze the performance. By expanding Eq. (B,5) in Taylor series up to the second order in Δt and then in Δx, we obtain

$$\begin{aligned}
\frac{\partial f}{\partial t}\Delta t &\approx f(x, t+\Delta t) - f(x,t) \\
&= -f + \sum_{j=1}^{2}\left(\pi_j^+ - \frac{\partial \pi_j^+}{\partial x}\Delta_j + \frac{1}{2}\frac{\partial^2 \pi_j^+}{\partial x^2}\Delta_j^2\right)\left(f - \frac{\partial f}{\partial x}\Delta_j + \frac{1}{2}\frac{\partial^2 f}{\partial x^2}\Delta_j^2\right) \\
&\quad + \sum_{j=1}^{2}\left(\pi_j^- + \frac{\partial \pi_j^-}{\partial x}\Delta_j + \frac{1}{2}\frac{\partial^2 \pi_j^-}{\partial x^2}\Delta_j^2\right)\left(f + \frac{\partial f}{\partial x}\Delta_j + \frac{1}{2}\frac{\partial^2 f}{\partial x^2}\Delta_j^2\right) + O\left(\Delta x^3\right) \\
&= -f + \sum_{j=1}^{2}\left[\left(\pi_j^+ + \pi_j^-\right) f - \left(\frac{\partial(\pi_j^+ - \pi_j^-)}{\partial x} f + \frac{\partial f}{\partial x}(\pi_j^+ - \pi_j^-)\right)\Delta_j\right] \\
&\quad + \sum_{j=1}^{2}\frac{\Delta_j^2}{2}\left[\frac{\partial^2(\pi_j^+ + \pi_j^-)}{\partial x^2} f + 2\frac{\partial(\pi_j^+ + \pi_j^-)}{\partial x}\frac{\partial f}{\partial x} + \frac{\partial^2 f}{\partial x^2}(\pi_j^+ + \pi_j^-)\right] + O\left(\Delta x^3\right).
\end{aligned} \tag{B,8}$$

Then, we neglect the third order of Δx and obtain

$$\frac{\partial f}{\partial t} = -\frac{\partial}{\partial x}\left(b(x) f\right) + \frac{1}{2}\frac{\partial^2}{\partial x^2}\left(a(x) f\right), \tag{B,9}$$

where b and a are, respectively, the drift and diffusion coefficients. Using Eq. (4) in the main text for the derivation of b, we obtain

$$b(x) = \sum_{j=1}^{2}\left(\pi_j^+ - \pi_j^-\right)\frac{\Delta_j}{\Delta t} \approx \frac{(P_1 - P_2)v}{2 - (P_1 + P_2)}, \tag{B,10}$$

$$\begin{aligned}
a(x) &= \sum_{j=1}^{2}\left(\pi_j^+ + \pi_j^-\right)\frac{\Delta_j^2}{\Delta t} \\
&= 2D\left\{\left[P_1 + \Omega^2(1-P_1)\right] P_{CW}(x) + \left[P_2 + \Omega^2(1-P_2)\right](1 - P_{CW}(x))\right\},
\end{aligned} \tag{B,11}$$

where $v = \Delta x/\Delta t$ and $D = 1/2\Delta x^2/\Delta t$, and Δx and Δt are kept as small but finite values. (When $\Delta x, \Delta t \to 0$, the coefficients b and a require the condition of $\sum_j(\pi_j^+ - \pi_j^-) = O(\Delta x)$, i.e., $|P_1 - P_2| \ll 1$, for consistency.)

The probability $f(x,t)dx$ is obtained by solving Eq. (B,9) from the initial state $f(x,0) = \delta(x)$ at $t = 0$ when there is no information on two machines. However, it is generally difficult to analyze the time evolution of f. Instead, we consider the time evolution of the mean of x, $\langle x \rangle = \int x f dx$, by assuming $f(x,t) \to 0$ and zero probability flux $F \to 0$ in the limit of $x \to \pm\infty$, and obtain the following results:

$$\frac{d\langle x \rangle}{dt} = \int x \frac{\partial}{\partial t} f(x,t)dx = \int F dx \approx \frac{(P_1 - P_2)v}{2 - (P_1 + P_2)}, \tag{B,12}$$

where F represents the probability flux,

$$F(x,t) = b(x)f - \frac{1}{2}\frac{\partial}{\partial x}\left[a(x)f\right]. \tag{B,13}$$

Then we obtain

$$\langle x \rangle_t \approx \frac{(P_1 - P_2)v}{2 - (P_1 + P_2)} t. \tag{B,14}$$

Obviously, for $P_1 > P_2$, $\langle x \rangle$ is shifted in the range of $x > 0$, whereas $\langle x \rangle$ is shifted in the range of $x < 0$ for $P_1 < P_2$. This suggests that $f(x,t)$ is mainly localized in the range of $x > 0$ ($x < 0$) for a large t when $P_1 > P_2$ ($P_1 < P_2$), and thus, $\mathrm{CDR}_{1(2)}$ monotonically increases.

Then, we consider such a function $P_{CW}(x)$ that the $\mathrm{CDR}_{1,2}$ is maximized. From Eqs. (B,6) and (B,7), the $\mathrm{CDR}_{1(2)}$ is approximately expressed for $\Delta x, \Delta t \ll 1$ as follows:

$$\mathrm{CDR}_1(t) = \int P_{CW}(x) f(x,t)dx, \qquad \text{and} \qquad \mathrm{CDR}_2(t) = \int \left[1 - P_{CW}(x)\right] f(x,t)dx. \tag{B,15}$$

When $P_{CW}(x)$ is always 1 for $\forall x \in (-\infty, \infty)$, the CDR_1 is maximized to be 1 because of $\mathrm{CDR}_1 = \int f dx = 1$ in this case. However, obviously, CDR_2 becomes zero when $P_1 < P_2$. Otherwise, when $P_{CW}(x) = 0$, CDR_1=0 for $P_1 > P_2$ but CDR_2=1 for $P_1 < P_2$. Therefore, to maximize both CDR_1 and CDR_2, $P_{CW}(x)$ should have a symmetry with respect to $x = 0$, $P_{CW}(x) = 1 - P_{CW}(-x)$. Moreover, $P_{CW}(x)$ should be 1 for $x > 0$ and 0 for $x < 0$, and steeply vary from 0 to 1 around $x \approx 0$, i.e., $dP_{CW}/dx|_{x=0} \gg 1$, considering that f is mainly localized in the range of $x > 0$ ($x < 0$) when $P_1 > P_2$ ($P_1 < P_2$).

$dP_{CW}/dx|_{x=0} \gg 1$ is important for adaptive and fast decision-making because the initial increasing rate of CDR, $d\mathrm{CDR}/dt|_{t=0}$, depends on dP_{CW}/dx as follows:

$$\left.\frac{d}{dt}\mathrm{CDR}_{1(2)}\right|_{t=0} = \pm \int \left.\frac{dP_{CW}}{dx}\right|_{x=0} F(0,t)dx \approx \frac{|P_1 - P_2|v}{2 - (P_1 + P_2)} \left.\frac{dP_{CW}}{dx}\right|_{x=0}. \tag{B,16}$$

For the derivation, we used $f(x,0) = \delta(x)$ and $P_{CW}(x) = 1 - P_{CW}(-x)$. The initial increasing rate can be improved by increasing $dP_{CW}/dx|_{x=0}$.

As shown in Fig. 5 in the main text, the $P_{CW}(x)$ designed such that the above conditions are satisfied certainly results in adaptive and correct decision-making when prior knowledge of $P_1 + P_2$ is provided. However, for the decision-making from zero knowledge, i.e., outside the random-walk model approximation, the designed $P_{CW}(x)$ is not always effective for improving the performance. A further investigation on the decision-making in general situations is an important future study.
